# The Relationship of Opium Addiction with Coronary Artery Disease

**Published:** 2010

**Authors:** Mohammad Masoomi, Mohammad Arash Ramezani, Hadi Karimzadeh

**Affiliations:** 1 Associate Professor of Cardiology, Department of Cardiology, Kerman University of Medical Sciences, Kerman, Iran; 2 MPH, Head of Surveillance Department, Isfahan Cardiovascular Research Center, Isfahan University of Medical Sciences, Isfahan, Iran; 3 General Practitioner

**Keywords:** Opium, Coronary artery disease, Angiography, Risk factor

## Abstract

**Objectives:**

There is some controversy regarding the effect of opium addiction on the coronary artery disease (CAD). The aim of this study was to determine the association between chronic opium consumption and CAD.

**Methods:**

This study had a case-control design. The patients recruited to the study were selected from angiography files in Department of Cardiology in Kerman University of Medical Sciences, Kerman, Iran. The comparison was done between CAD patients and normal subjects. Opium addiction was diagnosed by patient self-report and confirmed with interview based on DSM-IV criteria. Odds ratio with 95% confidence interval were estimated by unconditional logistic regression.

**Results:**

The risk factor of CAD was the same in the two study groups. The significant difference in opium consumption was demonstrated between CAD patients and normal coronary artery subjects (OR=3.8, 95%CI=1.5-9.5). Because of the strong association between cigarette smoking and opium addiction, analysis was done in smoker and non-smoker groups separately. Logistic regression showed opium addiction was the independent risk factor for CAD in non-smokers after adjusting to other CAD risk factors (OR=38, 95%CI=2.7-531.7), but in cigarette smokers opium was not a significant risk factor (OR=13.2, 95%CI=0.85-206.5).

**Conclusions:**

We confirmed that the opium was an independent risk factor for CAD. Health managers and policy makers should try to aware general population and prepare many preventive programs against substance abuse.

## INTRODUCTION

Primary investigation on morphine and its derivatives had been focused on central nervous system (CNS), thoroughly. It has been determined that opioids have many effects on different physiological systems. In recent years, there are a great deal of interest on cardiovascular system because of the cardioprotective role of opioid endogenous and exogenous compounds on ischemic preconditioning (IPC).^1^ Opioids influence three kinds of receptors in myocytes named mu (μ), kappa (κ) and delta (δ).^1^,^2^ It seems the cardioprotection in ischemic preconditioning is related to (κ) and (δ) receptors.^3^,^4^. Opioids cause IPC and attenuate ischemic reperfusion injury and decrease apoptosis in myocytes, thus infarct size is smaller and ventricular function ameliorates.^5^ On the other hand, we confront to substance abuse phenomenon. In a 20-year prospective study, the most common cause of death in opioid addicts was coronary artery disease (CAD) diseases (CAD), except accidental events. Furthermore, standardized mortality ratios for CAD has been higher in opioid addicts compared to general population.^6^ Opium abuse is a major predicament in many countries particularly in the Middle East region. In Iran, the opium addiction is predominant and its prevalence estimated over 3% in general population.^7^,^8^ The most frequent consumption method is inhalation.^9^ Although opioids have the protective effects on heart and play an important treatment role during ischemic event, the chronic usage of opioid may complicate influence of opioids^2^, particularly when the consumption ways are different like inhalation. Now, the important question is as follows: what is the effect of opium addiction on CAD? The previous investigation from our center revealed that the opium addiction didn’t correlate to myocardial infarction (MI).^10^ Another report from Iran determined that the opium addiction was a significant risk factor for CAD (OR=1.8, 95%CI=1.1-3.1).^11^ In contrast, Marmor and colleagues reported that CAD prevalence in methadone or opioid addict decedents was less than that in non-addict ones and even opioid addiction had a protective effect on CAD (OR=0.43, 95%CI= 0.20 to 0.94).^12^ This dilemma motivated us to investigate the association of opium addiction and CAD. The aim of this study was to determine the association between opium addiction and CAD by a different design.

## MATERIALS AND METHODS

### 

#### Setting

The study was conducted in Department of Cardiology and Angiography in Shafa Medical Center, one of the biggest hospitals of Kerman University of Medical Sciences. The angiography ward is the only cath lab in Kerman province. We carried out a cohort study of chest pain in our department since February 1^st^ 2007. It included 137 cases till February 1^st^ 2008. The nested case-control study was designed in the cohort study. We recruited the patients who were catheterized. Those cases with positive findings in catheterization were selected as case group and subjects with normal angiography were included in the control group. The research proposal was approved by both research and medical ethics committees in Kerman University of Medical Sciences.

#### Data collection

Demographic and cardiovascular risk factor information (including hypertension, hyperlipidemia, diabetes mellitus, family history of CAD and smoking) and angiographic data were collected by reviewing the patients’ records. Besides, opium addiction was diagnosed by patient self-report and confirmed with interview based on DSM-IV criteria. Type of substance abuse, duration of abuse and kind of consumption were recorded in interview. All data were registered in structured data collection forms.

#### Statistical analysis

Data was compared between the two CAD positive and negative groups. The chi-square test was used for analysis of nominal variables. The mean was compared with t test and when it was ranked, the Mann-Whitney test was appropriated. Odds ratio and confidence interval was calculated for every risk factor and tested with Mantel-Haenszel test. The roles of established CAD risk factors as effect modifiers or confounders were evaluated by stratification and were finally considered in the logistic regression models. The analyses were performed by using the SPSS release for Windows, Version 15 (SPSS Inc., Chicago, IL). The P<0.05 was considered significant.

## RESULTS

**Figure 1:**
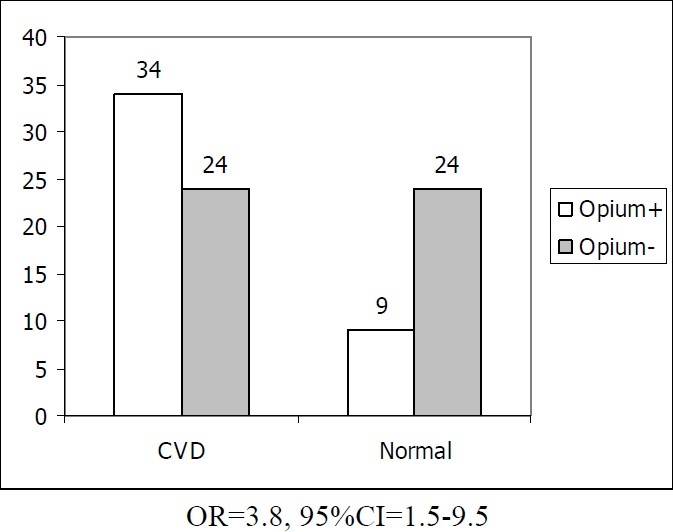
The comparison of opium addiction between the cardiovascular disease patients and normal groups.

Of 137 cases in our cohort, 91 patients were catheterized and recruited. CAD was delineated in 58 (63.7%) patients; 33 (36.3%) had normal angiography.Table 1shows the demographic characteristics in the two groups. As shown in theTable 1, there were any significant differences in demographic and risk factors between the two study groups. The frequency of opium addiction was 3.8 folds greater in CAD patients and the difference was significant. The significant difference in opium consumption was shown in Figure 1.

**Table 1:** The frequency of demographic variables and risk factors in the two study groups.

Variables	CVD+	CVD-	P value
**Sex**			
Male	47	22	0.136*
Female	11	11	
**Age (mean ± SD)**	57 ± 3.4	57.1 ± 3.1	0.848†
**Hypertension**			
Positive	17	8	0.635*
Negative	41	25	
**Diabetes**			
Positive	12	6	0.99*
Negative	46	27	
**Hyperlipidemia**			
Positive	19	9	0.643*
Negative	39	24	
**Family history**			
Positive	26	9	0.12*
Negative	32	24	
**Smoking**			
Positive	24	12	0.663*
Negative	34	21	

* *Fisher’s exact test

† independent sample t-test

The risk factors and demographic characteristics were the same in addicted CAD patients and those with normal coronary artery (Table 2). The method of consumption and duration of abuse demonstrated no differences between CAD and normal cases. Additionally, in CAD cases the angiographic characteristics like number of vessels suffering from atherosclerosis, severity of obstruction and ejection fraction didn’t differ between addicted and non-addicted patients. Notably, the strong association between cigarette smoking and opium consumption not only existed in CAD patients, also was observed in healthy subjects (Table 3). Just as coming from tables 1, 2 and 3, the conventional risk factors of CAD correlated neither dependent nor independent variables. However, we modified the effect of cigarette smoking by stratification and multiple logistic regression analysis adjusted the effect of opium addiction by the other risk factors. As shown in table 4, opium abuse was independent risk factor for CAD in non-cigarette smoking cases (OR=38, 95%CI=2.7-531.7), but in cigarette smokers, opium was not a significant risk factor (OR=13.2, 95%CI=0.85-206.5).

**Table 2:** The frequency of risk factors in opium addicted patients.

Variables	CVD+	CVD−	P value*
**Hypertension**			
Positive	5	1	0.99
Negative	29	8	
**Diabetes**			
Positive	4	0	0.752
Negative	30	9	
**Hyperlipidemia**			
Positive	13	2	0.458
Negative	21		7	
**Family history**			
Positive	17	1	0.06
Negative	17	8	
**Smoking**			
Positive	2	4	0.666
Negative	22	20	

* *Fisher’s exact test

**Table 3:** Association between cigarette smoking and opium usage in the two groups under study.

	CVD+		CVD−	
	Opium+	Opium−	Opium+	Opium−
Smoking+	22	2	8	4
Smoking−	12	22	1	20
	OR=20.2, 95%CI=4−100.9		OR=40, 95%CI=3.8−415	

**Table 4:** Crude and adjusted odds ratio of CVD risk factors separated by cigarette smoking.

		CS+		CS−
	Crude OR (95%CI)	Adjusted OR (95%CI)	Crude OR (95%CI)	Adjusted OR (95%CI)
**Opium**	5.5 (0.8−36)	13.2 (0.8−206.5)	10.9 (1.3−91.6)	38 (2.7−531.7)
**Hypertension**	−	−	1 (0.3−3.1)	2 (0.5−8.6)
**Diabetes mellitus**	1 (0.15−6.4)	3 (0.2−42.8)	1.3 (0.34−5)	4.2 (0.7−24.9)
**Hyperlipidemia**	9.3 (1.03−84)	19.9 (0.9−442.9)	0.5 (0.15−1.6)	0.1 (0.02−0.9)
**Family history**	2.5 (0.5−11.8)	1.8 (0.25−12.8)	2 (0.6−6.3)	1.7 (0.4−6.6)

CS = cigarette smoking

## DISCUSSION

The results of current study revealed that the frequency of opium addiction in CAD patients was greater than that in normal ones (58.6% vs. 27.3%). However, after adjusting with other risk factors and stratifying based on cigarette smoking because of the great association between opium addiction and cigarette smoking, logistic regression model determined that in non-smokers opium addiction was the independent risk factor for CAD with OR=38 (95%CI=2.7-531.7). In contrast, although the OR was equal to 13.2 in smokers, the difference was not significant (95%CI=0.85-206.5). The opium addiction in our country, Iran, is a very dramatic public health problem. On the other hands, the mortality and morbidity due to CAD is located on the top of the mortality and morbidity list in Iran; many investigators attempt to find the association between opium, CAD and atherosclerosis phenomenon. Previous studies have shown that the range of opium addiction is varied from 10% in coronary artery bypass graft patients^13^ to 20% in patients with MI.^14^ One report from our center showed that the opium addiction was not a risk factor in MI patients.^10^ Sadeghian and colleagues excluded the cigarette smokers from their study and similar to our findings, the opium addiction was the independent risk factor for CAD (OR=1.8, 95%CI=1.1-3.1). They obtained information from angiographic records, retrospectively.^11^ The other study carried out in patients with cerebrovascular accidents declared the association of opium addiction and cerebrovascular stroke.^15^ Marmor and colleagues assessed the coronary arteries in decedents who exposed long-term to opiate. They found the opiate dependency was the protective factor for CAD (OR=0.43, 95%CI=0.2-0.94).^12^ While there is a clinical doubt regarding the relationship of opium addiction and CAD, the physiopharmacologic studies support the effect of opioid on cardiovascular system. Chronic consumption of opioid some effects on heart like release of endogenous substances like P, calcitonin genrelated peptide, adenosine and superactivate adenosine sensitivity and adenylyl cyclase that have the protective effect on cardiac function.^2^ However, the effect of opioid depends on the receptor subtype activation. For example, Aitchison et al. determined that the excitation of κ receptors deteriorates myocardial perfusion and increases the infarct size dramatically.^16^ Coles and colleagues reported that blockade of the κ opioid receptors abrogated reperfusion arrhythmias.^17^ It is interesting that morphine and its derivatives administration during cardiovascular events not only is not harmful also satisfies the patient and the cardiologist. However, chronic consumption of opiates as abuser may be an independent risk factor for CAD. The controversy exists in different studies due to some reasons. For example, opium abuse decreased the serum lipid level.^18^ But, the new risk factors of CAD vary in opium addicts. Glycosylated hemoglobin, lipoprotein A, C reactive protein, apo B, liver enzymes (ALT, AST) were significantly higher in addicts than in non-addicts; and apo A was lower contrarily.^19^ Additionally, method of opium consumption influences the lipid and the aforementioned new cardiovascular risk factors, in such a manner that morphine concentration was significantly higher in oral administration.^19^ A few people in Iran believe the protective effects of opium on cardiovascular disease or diabetes.^20^ As we demonstrated, opium is an independent CAD risk factor, thus public health manger and policy makers should plan many programs to elevate the knowledge, and finally, change the attitude and practice of general population regarding the opium consumption and its disadvantages. We had some limitations in our study. The best epidemiologic study to fit for the aim of current study was cohort and our design was case-control. However, it was a nested case-control in the cohort study and could confirm the association between opium addiction and CAD. Another limitation in our study was detection of addiction. We used verbal autopsy by aid of DSM-IV criteria and were not able to confirm the addiction by lab test. We endeavored to be accurate as much as possible.
